# An Estimate of Transfusion-Transmitted Infection Prevalence in General Populations

**DOI:** 10.5812/kowsar.1735143X.799

**Published:** 2011-12-20

**Authors:** Ahmad Gharehbaghian

**Affiliations:** 1 Medical Laboratory Sciences Department, School of Allied Medical Sciences, Shahid Beheshti University of Medical Sciences, Tehran, IR Iran; 2 Blood Transfusion Research Center, High Institute for research and education in Transfusion Medicine, Tehran, IR Iran

**Keywords:** Blood Transfusion, Populations

Dear Editor,

Blood donations saves the lives of millions of people worldwide, and blood transfusion is essential to the effectiveness of the health care system by supporting modern medicine as its pivotal role in patient interventions [1][2]. New, more sophisticated medical and surgical procedures such as transplant, heart surgery, and trauma or cancer treatment depend highly on blood transfusions in each country. Moreover, blood samples improve the quality of life of multitransfused patients. In developing countries transfusion-transmitted infections (TTIs) often threaten the safety of patients requiring blood transfusion, and medical providers face serious challenges with blood availability, safety, and affordability. It is estimated that 45% of the 80 million blood donations across the globe are collected each year in developing countries that comprised roughly 80% of the world’s population [3][4].

On a national level, countries should secure the safety of patients requiring blood transfusion by creating standard blood-transfusion establishments with solid support from policy makers and adequate and consistent government funding. One of the most prominent factors in ensuring safe blood samples is to have a national program for donor selection, recruitment, retention, and education to minimize donations from donors who might transmit diseases to recipients [2][4]. In addition, to ensure blood safety it is important to monitor and evaluate TTIs, including hepatitis B virus (HBV) and hepatitis C virus (HCV), among blood donors and in the general population. The prevalence of transfusion transmitted infections among blood donors in well-structured health care system with a well-organized blood establishment can be used as a reliable tool for statistical estimations of those infectious agent that can be transmitted through blood products including Hepatitis B and C. Indeed, the prevalence size of HCV or HBV among blood donors in such countries can contribute to statistical estimation of those viruses in the general population[5]. In Petrovic’s study, the prevalence rates of chronic HBV and HCV in the general populations of the Tuzla Canton in Bosnia and Herzegovina have been estimated by the adjusted prevalence of HBs-Ag and anti-HCV seropositive samples among first-time blood donors. In the current study the statistical analysis of prevalence rates has been performed based on incidence calculation of the estimated ratio between the two populations (the first-time blood donors and general population) using Poisson distribution method through reviewing published papers[6].

However, epidemiological studies of HBV and HCV in general populations have faced difficulties in obtaining sufficient sample sizes, gender and age distributions, and financial support. Nevertheless, the process of blood-donor selection aims to identify and recruit nonremunerated, apparently healthy people willing to donate blood. The donated blood should be free of bloodborne infectious agents that can be transmitted to recipients, and this depends highly on the structure of the country’s health care system [6][7]. In addition, blood-transfusion establishments should meet the international requirements for standard operating procedures that play a pivotal role in protecting the safety of both donors and recipients. Especially after the AIDS epidemic, the concern about blood safety in multitransfused patients has increased greatly. As the authors have explained, recruited blood donors are usually healthier because they are from people in the community who tend to exhibit healthier behaviors and safer life styles[8].

In conclusion, the accuracy of estimating TTI prevalence in the general population by surveying first-time blood donors depends on how similar the demographic characteristics of the two populations are. What we perceive from this study are significant differences in TTI prevalence rates between first-time and regular blood donors (data not shown here but available upon request), particularly in terms of demographic characteristics (e.g., cultural-economic issues), the structure of regional or country health care systems, blood transfusion service structures, blood donation incentives, blood type of donors (paid-donors, family replacement donors, or volunteer nonremunerated blood donors). Indeed, it seems that the populations of the countries or regions in which the reviewed studies were conducted are not demographically compatible or similar enough for valid inferences to be made.

## References

[1] Eyles J, Heddle N, Webert K, Arnold E, McCurdy B (2011). Do expert assessments converge? An exploratory case study of evaluating and managing a blood supply risk.. BMC Public Health.

[2] Gharehbaghian A, Abolghasemi H, Namini MT (2008). Status of blood transfusion services in Iran.. Asian J Transfus Sci.

[3] Reynolds L, McKee M (2010). Matching supply and demand for blood in Guizhou province, China: an unresolved challenge.. J Public Health (Oxf)..

[4] World Health Organization;Blood transfusion safety.

[5] Omidkhoda A, Gharehbaghian A, Jamali M, Ahmadbeigi N, Hashemi SM, Rahimi A, Soleimani M (2011). Comparison of the prevalence of major transfusion-transmitted infections among Iranian blood donors using confidential unit exclusion in an Iranian population: Transfusion-transmitted infections among Iranian blood donors.. Hepat Mon.

[6] Petrovic J, Salkic NN, Ahmetagic S, Stojic V, Mott-Divkovic S (2011). Prevalence of Chronic Hepatitis B and Hepatitis C among First Time Blood Donors in Northeast Bosnia and Herzegovina: An Estimate of Prevalence in General Population.. Hepat Mon.

[7] Ismail AM, Devakumar S, Anantharam R, Fletcher GJ, Subramani T, John GT, Daniel D, Abraham P (2011). Low frequency of occult hepatitis B infection in anti-HBc seropositive blood donors: experience from a tertiary care centre in South India.. Blood Transfus.

[8] Cheraghali AM, Gharehbaghian A (2011). Commentary on: Effectiveness of confidential unit exclusion option in blood transfusion services needs re-evaluation and Author's reply.. Hepat Mon.

